# First‐of‐its‐kind identification of two novel tau mutations associated with cognitive resilience to Alzheimer’s disease neuropathology

**DOI:** 10.1002/alz.095561

**Published:** 2025-01-09

**Authors:** Danielle Jamison, Giulio Taglialatela

**Affiliations:** ^1^ University of Texas Medical Branch, Galveston, TX USA

## Abstract

**Background:**

To date, mutations in the MAPT (i.e., tau) gene within Alzheimer’s disease (AD) patients are either benign or have an unclear effect on pathogenicity. Significant prior literature and evidence from other tauopathies support the hypothesis that mutations can alter tau’s structure and function. Approximately one‐third of neuropathological autopsies identify individuals with comparable AD neuropathology (i.e., extracellular amyloid‐beta (Aβ) plaques and intracellular tau‐containing neurofibrillary tangles (NFTs)) without similar clinical manifestations or cognitive deficits (herein referred to as non‐demented with Alzheimer’s neuropathology or NDAN). The comparable neuropathological burden with divergent clinical manifestations between AD patients and NDAN individuals led us to hypothesize that a mutation in the NDAN MAPT gene could explain, at least in part, this dichotomous presentation.

**Method:**

RNA was isolated from the frontal cortices of seven NDAN individuals (4M, 3F) using a commercial RNA kit. Primers to amplify the tau coding sequence were found in the literature and confirmed to amplify the tau coding sequence. With assistance from UTMB’s Next Generation Sequencing core, the RNA quality was first assessed, and subsequently, library generation and sequencing were performed.

**Result:**

Two novel somatic mutations were identified in all seven individuals with frequencies consistent with mosaic mutant frequencies. The first, an aspartic acid to alanine mutation, occurred at a position where no prior mutations have been documented. The second occurs at the serine immediately preceding tau’s amyloid motif, VQIVYK; in this mutation, the phosphoresidue is replaced by a charged and bulky arginine. Analysis using the Ensembl Variant Effect Predictor (VEP) predicted both mutations to be, at a minimum, moderately damaging to tau’s function, which is logical considering the amino acid substitutions occurring. The severity of each mutation’s effect on the tau structure and function was isoform specific.

**Conclusion:**

We identified, to the best of our knowledge, two completely novel tau mutations uniquely associated with individuals that remain cognitively intact despite abundant AD neuropathology. These mutations may help contextualize the unique phenotype observed in NDAN individuals, such as cognitive resilience and spreading with minimal toxicity.

**Funding Support**: R01422460 and R0142690 to GT. Attendance at this conference was supported by an AAIC 2024 Conference Fellowship.

